# Amide hydrogens reveal a temperature-dependent structural transition that enhances site-II Ca^2+^-binding affinity in a C-domain mutant of cardiac troponin C

**DOI:** 10.1038/s41598-017-00777-6

**Published:** 2017-04-06

**Authors:** Tiago Veltri, Guilherme A. P. de Oliveira, Ewa A. Bienkiewicz, Fernando L. Palhano, Mayra de A. Marques, Adolfo H. Moraes, Jerson L. Silva, Martha M. Sorenson, Jose R. Pinto

**Affiliations:** 1 Department of Biomedical Sciences, Florida State University College of Medicine, 1115 West Call Street, Tallahassee, FL 32306-4300 USA; 2 Instituto de Bioquímica Médica, Universidade Federal do Rio de Janeiro, Av. Carlos Chagas F° 373, Cidade Universitária, Rio de Janeiro, 21941-902 RJ Brazil; 3 Programa de Biologia Estrutural, Instituto de Bioquímica Médica, Instituto Nacional de Biologia Estrutural e Bioimagem, Centro Nacional de Ressonância Magnética Nuclear Jiri Jonas, Universidade Federal do Rio de Janeiro, Rio de Janeiro, Brazil; 4 Departamento de Química, Instituto de Ciências Exatas, Universidade Federal de Minas Gerais, Brazil

## Abstract

The hypertrophic cardiomyopathy-associated mutant D145E, in cardiac troponin C (cTnC) C-domain, causes generalised instability at multiple sites in the isolated protein. As a result, structure and function of the mutant are more susceptible to higher temperatures. Above 25 °C there are large, progressive increases in N-domain Ca^2+^-binding affinity for D145E but only small changes for the wild-type protein. NMR-derived backbone amide temperature coefficients for many residues show a sharp transition above 30–40 °C, indicating a temperature-dependent conformational change that is most prominent around the mutated EF-hand IV, as well as throughout the C-domain. Smaller, isolated changes occur in the N-domain. Cardiac skinned fibres reconstituted with D145E are more sensitive to Ca^2+^ than fibres reconstituted with wild-type, and this defect is amplified near body-temperature. We speculate that the D145E mutation destabilises the native conformation of EF-hand IV, leading to a transient unfolding and dissociation of helix H that becomes more prominent at higher temperatures. This creates exposed hydrophobic surfaces that may be capable of binding unnaturally to a variety of targets, possibly including the N-domain of cTnC when it is in its open Ca^2+^-saturated state. This would constitute a potential route for propagating signals from one end of TnC to the other.

## Introduction

Cardiac troponin C (cTnC) controls key events of systole and diastole through its ability to bind and release Ca^2+^, and changes in its Ca^2+^ affinity can promote a systolic or diastolic dysfunction that leads to a chronic problem, typically followed by remodeling of the ventricular wall. cTnC has two globular domains connected by a long flexible linker and three EF-hand sites for Ca^2+^ binding; when the TnI switch peptide binds, the B and C helices in the cTnC N-domain swing out away from the others to accommodate it^1, 2^. The N-domain of cTnC has only one functional site (site II), where Ca^2+^ triggers conformational changes that lead to adjustments in the thin-filament proteins to permit actin-myosin interaction^3^. The C-domain anchors cTnC to the thin filament, and binds Ca^2+^/Mg^2+^ competitively at sites III and IV. It is now generally accepted that events in one domain can have an effect on the other, especially when the other proteins of the thin filament are present, but the mechanism is not clear^4–6^.

Over the last two decades, mutations in sarcomeric proteins that lead to cardiomyopathies have been studied extensively^7^. Recently, four new mutations related to familial hypertrophic cardiomyopathy (HCM) were discovered in the human *TNNC1* gene^8^. Among these, the D145E proband had a positive family history for HCM, suggesting segregation of the mutation among family members as the cause. The mutation D145E is located in the C-domain of human cTnC (HcTnC), where it drastically reduces Ca^2+^-binding affinity^9^. Its capacity for causing diastolic dysfunction has been attributed to its effect at the N-domain site II, where it conversely increases the affinity for Ca^2+^ and consequently delays cardiac muscle relaxation^8, 10^. This mutation also causes minor changes in certain structural parameters, including the α-helical content^11^.

In troponin and tropomyosin, most mutations that are related to HCM tend to increase Ca^2+^ sensitivity of the thin filaments (for review, see ref. 12), and HcTnC D145E is no exception. However, the mechanism by which a disease-associated mutation in the TnC C-domain can increase Ca^2+^ affinity in the N-domain is unknown. Since Ca^2+^ binding to cTnC leads to global structural changes^13, 14^, we postulated that stability and folding might be disrupted, and we sought to correlate the stability of the HcTnC D145E with its physiological function in skinned fibres. For skeletal TnC, several reports relate protein folding and function^15, 16^, whereas for cardiac TnC, folding and stability^6^ have attracted less attention than quaternary structure^17^. Here we used nuclear magnetic resonance (NMR) of the recombinant HCM protein to obtain the backbone assignment, and we evaluated its stability and structural features by NMR and circular dichroism (CD) at different temperatures. In a separate report, we have looked for evidence of changes in the internal dynamics of the N- and C-domains of the mutants^18^.

Since in these experiments temperatures near the physiological range promoted substantial changes in HcTnC D145E structure that were not seen in the WT protein, we also investigated the effect of temperature on the function of HcTnC D145E. For both skeletal and cardiac muscle, it is well known that changes in temperature alter force development, Ca^2+^ sensitivity and the rate of sarcomere shortening^19, 20^. Thus, a second aim was to evaluate the temperature dependence of stability and function for the HcTnC D145E mutant incorporated into skinned cardiac myofibrils and compare it with the WT protein using temperatures in the near-physiological range.

## Results

### Residues affected by the D145E mutation

**Figure 1:**
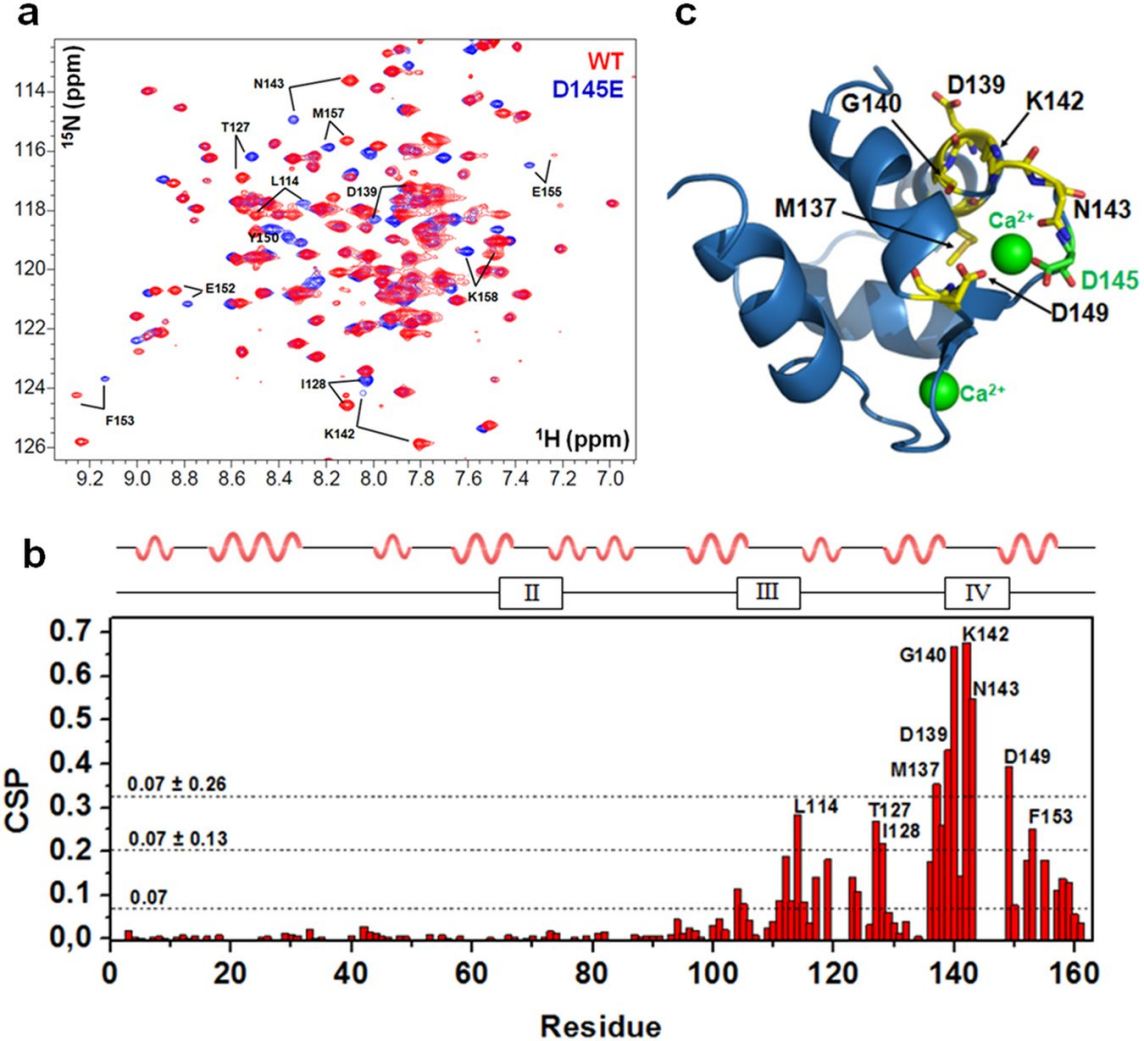
Mutation D145E affects the chemical environment. (**a**) Superposition of ^1^H-^15^N-HSQC NMR spectra for Ca^2+^-bound HcTnC WT (red) and D145E (blue) reveals the most perturbed residues in the D145E protein at 25 °C. (**b**) Chemical-shift perturbation (CSP) plots for the N- and C-domains as a function of residue. Dashed lines show the average and one and two s.d. above the average. Residues with CSP values ≥ 2 s.d. are shown by yellow backbones on HcTnC in **c**, the WT NMR structure (PDB: 1AJ4) for the C-domain. Ca^2+^ ions are shown as green spheres and D145 as a green stick.

Although several backbone assignments are available for WT cardiac troponin C in the Biological Magnetic Resonance Data Bank^21, 22^, none matches exactly the entire primary sequence and buffer conditions used in this work. Thus, we recorded a complete experimental set of triple-resonance NMR spectra for HcTnC WT and D145E backbone assignments. Structural changes in HcTnC caused by the D145E mutation were identified by mapping the chemical-shift perturbations (CSP) between the ^1^H-^15^N HSQC spectra of WT and D145E at 25 °C (Fig. 1a,b). CSP data revealed that incorporation of glutamate at position 145 perturbed almost all residues of HcTnC C-domain, with a more pronounced effect for residues located closer to the mutation site (Fig. 1b). Residues 144–148 could not be assigned because of the disappearance of the peaks. Perturbed residues (deviating by ≥ 2 s.d. from the average) are highlighted in the image of the Ca^2+^-bound protein (PDB: 1AJ4) (Fig. 1c), showing that this HCM-related mutation in Ca^2+^-binding site IV strongly affects residues in its vicinity. In the N-domain, CSP perturbations were vanishingly small, and so we turned to other techniques for closer analysis.

### HcTnC thermostability

**Figure 2:**
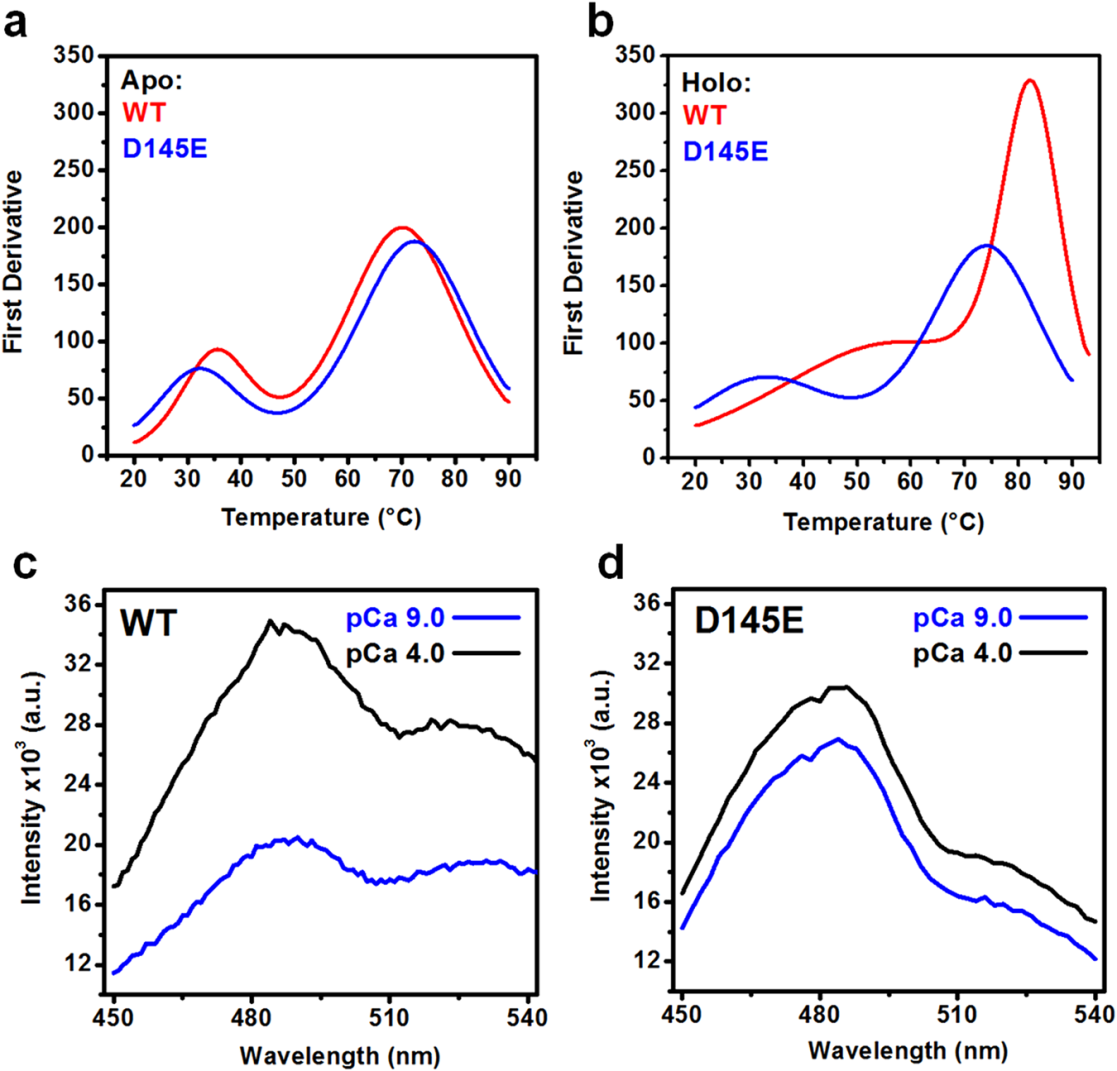
Thermostability and hydrophobic exposure of WT and D145E. Mean residue ellipticity at 222 nm was measured for HcTnC WT and D145E. Data shown are sample derivatives from 3 experiments with 2 different protein batches in apo **(a**) and Mg^2+^/Ca^2+^-bound (**b**) states. All melting curves were run in reverse after reaching 90 °C, and the structural changes promoted by temperature were reversible. For average values, see text. In (**c**), 5 μM bis-ANS was excited at 360 nm in the presence of 1 μM WT or D145E to compare fluorescence intensity at low (pCa 9.0) and high (pCa 4.0) Ca^2+^ concentrations as a measure of binding to hydrophobic surface areas. Average ratios (holo/apo ± s.e.m.) were 1.68 ± 0.11 for WT and 1.13 ± 0.02 for D145E (p = 0.007).

Melting curves measured using circular dichroism (shown as first derivatives) for WT and D145E proteins in EGTA (apo state) were not statistically different (Fig. 2a), with two transitions and temperature at the midpoint of transition (T_m_) values of 36.6 ± 1.5 and 70.9 ± 0.6 *vs* 32.9 ± 0.5 and 72.5 ± 0.4 °C, respectively (mean ± s.e.m., n = 3). The second T_m_ values were very similar to data recorded for the isolated WT N-domain^23^ as well as for the full-length protein fitted to a single-transition equation^24^. With Ca^2+^/Mg^2+^ present (holo state), however, the two proteins diverged: the WT became very stable, with a single transition at a higher temperature (T_m_ 81.8 ± 0.6 °C), while the mutant essentially reproduced the result recorded in EGTA (T_m1_ 32.9 ± 0.5 and T_m2_ 72.5 ± 0.4 °C) (Fig. 2b). We know that Ca^2+^ binds to the N-domain of D145E; thus the melting that occurs with the mutant appears to be a property of the C-domain, where Ca^2+^ does not appear to enhance the stability.

### Hydrophobic interactions

In a further attempt to evaluate overall structural changes in the mutant, we recorded the fluorescence of 4,4′-dianilino-1,1′-binaphthyl-5,5′-disulfonic acid (bis-ANS) as a measure of binding of this probe to hydrophobic surfaces on HcTnC. The fluorescence of bis-ANS in our experiments increased by 68% upon conversion of the WT TnC from apo to holo state (Fig. 2c), indicating additional exposure of hydrophobic surface area on binding Ca^2+^. However, the finding of similar signals from the mutant regardless of Ca^2+^ (*cf*. pCa 9 *vs*. pCa 4 in Fig. 2d) showed us that its C-domain was already in an open conformation without Ca^2+^. It is also possible that it remains closed, but distorted toward unusual hydrophobic exposure in both the presence and absence of Ca^2+^.

### Secondary-structure analysis at room temperature

**Figure 3:**
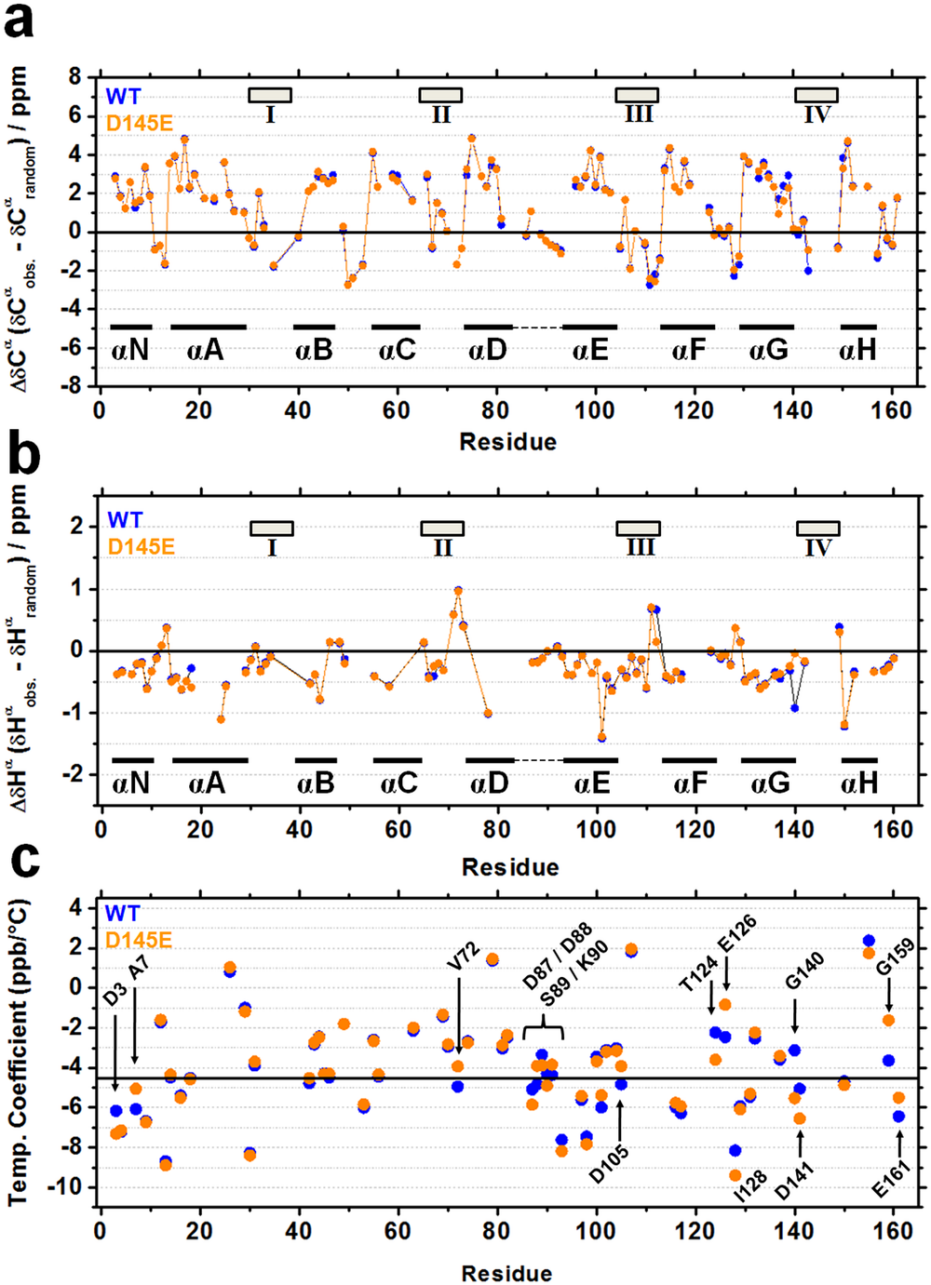
Secondary structure at 25 °C (**a**,**b**) and distribution of amide hydrogen temperature coefficients over the range 15°–55 °C (**c**) among WT () and D145E () residues. Deviation of experimental (obs.) chemical shifts (δ) from random coils for backbone C^α^ (a) and H^α^ (**b**). Random-coil chemical shift values were obtained from ref. 27. Rectangles (I–IV) and black line segments (αN, αA, etc) identify Ca^2+^-binding sites and α-helices known to be present in WT cTnC. Alpha-helical segments show positive deviations for C^α^ and negative deviations for H^α^, while β-strands and loops show the opposite. Dashed lines between α-D and α-E in **a** and **b** represent the D/E linker region. In (**c**), amide hydrogens with temperature coefficients that were linear but very different (>95% C.I.) for WT and mutant proteins are identified by residue. Temperature coefficients of the protons coincide (or differ only slightly) for the two isoforms except where one of the paired values is labeled with an arrow.

In a previous report, the circular dichroism data for the mutant showed small changes in ellipticity (~5%) compared to the WT protein^11^. To probe more accurately for possible changes in secondary structure, based on NMR CSPs we compared deviations of α-carbons (C^α^) and α-protons (H^α^) in the backbone of each protein with values for the same residues in a set of model random-coil peptides^25–27^. The expected result of this residue-by-residue analysis is that α-helical segments show positive deviations for C^α^ and negative deviations for H^α^, while β-strands and loops show the opposite. Overall, we observed significant differences between the WT and mutant secondary structure only in the G-helix (Fig. 3a and b), with little or no effect on residues of the N-domain. Thus the experiments of Figs 2, and 3a,b show that the site-IV mutation affects thermostability and tertiary structure but has very little impact on secondary structure, especially in the N-domain.

### Mutation effects on ^1^H^N^ temperature coefficients reveal alternative targets above 30–40 °C

Another clue to multiple changes in HcTnC caused by the D to E mutation in site IV emerges from measuring temperature coefficients (ppb/°C) for the chemical shift of the amide hydrogens (^1^H^N^) of each residue (*see Methods*). Temperature coefficients are inversely proportional to bond length, and a positive, linear coefficient denotes a simple process of thermal expansion, while a positive but non-linear coefficient can mean that a linkage is not only getting longer but is also sampling an alternative conformation^28, 29^. Although conformational changes, aromatic residues and other factors unrelated to hydrogen bonding can also alter amide temperature coefficients^28, 29^, values more negative than −4.5 ppb/°C are commonly involved in protein-solvent hydrogen bonds, while those less negative than −4.5 ppb/°C tend to be associated with intramolecular bonds, frequently observed in secondary contacts^28^. Additional experiments would be required to identify hydrogen bonds unambiguously. However, in this set of data, we were able to compare temperature coefficients of WT and D145E residues that displayed linear ^1^H shifts with temperature in {^1^H-^15^N} HSQC experiments (Fig. 3c). Three residues lying outside the 95% confidence limits for the 45° correlation line between these two sets of data are highlighted in Fig. 3c for the N-domain and eight are identified for the C-domain, out of a total of 59 with linear coefficients in both constructs. Interestingly, four of the outliers (D87–K90) are clustered in the linker between helices D and E, a region that is critical for Ca^2+^ sensitivity of the Tn complex and for proper regulation by the cTnI regulatory region (TnI_128–180_)^30^. These residues drew our attention because they appear to be strategically located for an impact on transmission of signals from C- to N-domain. Thus the D145E mutation impacts temperature coefficients throughout the C-domain and the inter-domain linker, but not in the N-domain, except for a few scattered residues.

**Figure 4:**
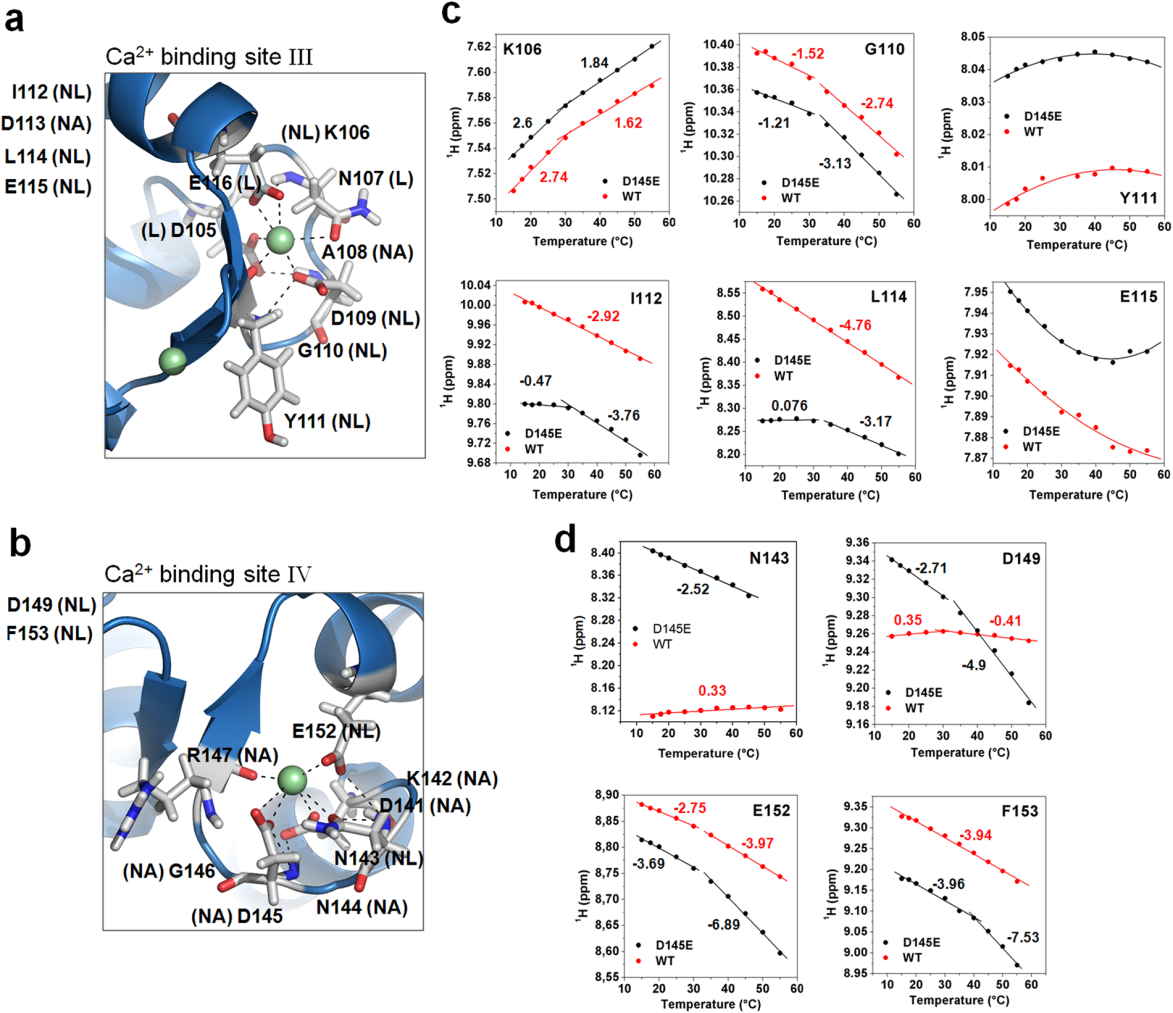
Residues with non-linear temperature coefficients for amide hydrogens are sampling a different chemical environment above the break. (**a**,**b**) Ca^2+^-binding sites III and IV reveal multiple residues with non-linear temperature dependence. Residues listed to the *left* of each panel are located before or after the cartoons in panels a and b (NL – nonlinear, L – linear and NA – not analysed). (**c**,**d**) Collection of ^1^H chemical shifts with nonlinear behavior for residues located within sites III and IV, respectively. Numbers beside line segments in (**c–d**) represent temperature coefficients in ppb/°C. Calcium ions (green) are depicted in both sites but available evidence indicates they are bound only weakly (or not at all) in sites III and IV of D145E.

Turning to residues where ^1^H^N^ signals underwent nonlinear shifts with temperature increase, we found that most of them were located within or near Ca^2+^-binding loops of the C-domain (Fig. 4a,b). These non-linear shifts in general depend on the equilibrium between two different conformations, but in these cases there may be a direct effect of Ca^2+^ on the observed chemical shifts, making them difficult to interpret. For the WT protein, 23 out of 110 cross-peaks analysed revealed nonlinear shifts and for D145E it was 27 out of 101. For many of these residues there was a linear shift with temperature from 15 °C up to a point near the physiological range (30 to 40 °C); at higher temperatures, the slope changed. Curves of this type were detected for residues in sites II, III and IV, with significant differences between WT and mutant found only in sites III and IV (Fig. 4c,d).

We found that temperature dependence for ^1^H^N^ chemical shifts of residues I112 and L114, located within Ca^2+^-binding site III but not participating directly in ion coordination, differed markedly in D145E compared to the WT protein, with a break point between 30 °C and 35 °C for D145E (Fig. 4c). At Ca^2+^-binding site IV, residues N143, D149, E152 and F153 also were very different for D145E compared to WT (Fig. 4d). We infer that D145E destabilizes Ca^2+^ binding to site IV, and this is manifested in more negative temperature coefficients, especially above 30 °C.

### Residue-specific changes with temperature

**Figure 5:**
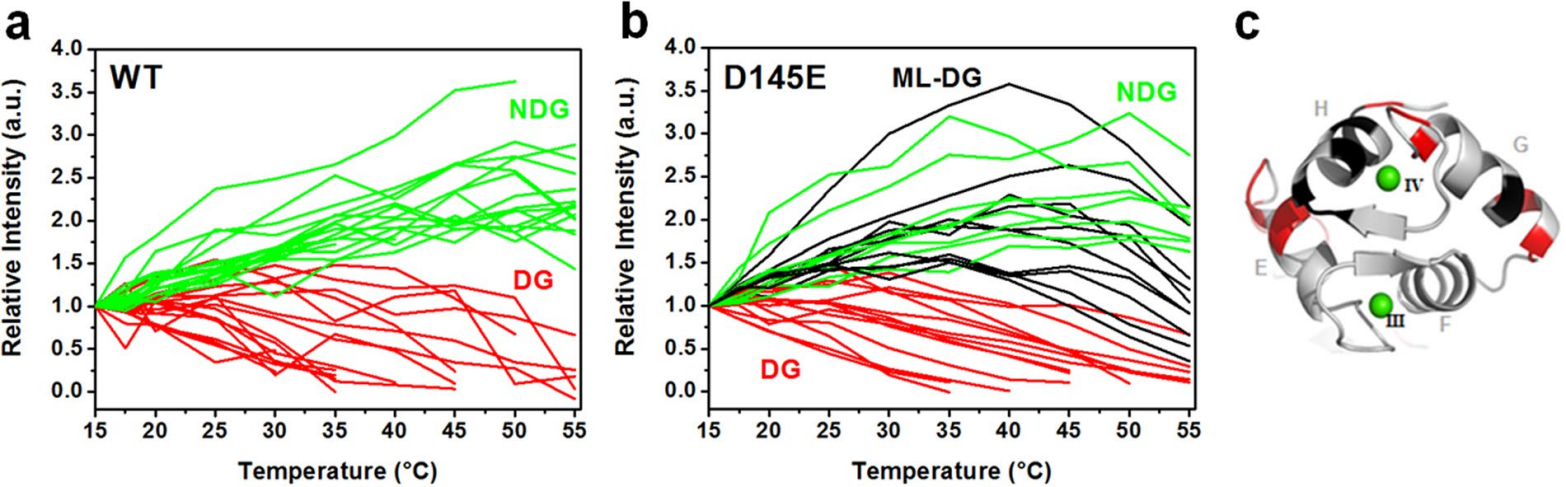
C-domain residues of WT and D145E isoforms differ in stability, as measured by NMR cross-peak intensities between 15° and 55 °C. (**a**,**b**) Changes in resonance intensities of the C-domain as a function of temperature, normalized to values at 15 °C. *Green* residue intensities are unperturbed or increased by temperature changes and classified as non-decaying group (NDG). *Red* residues lose intensity and are classified as decaying group (DG). In *Black* is the biphasic decaying group (“mountain-like”, ML-DG) for D145E. (**c**) Bottom view of the mutant cTnC C-domain, highlighting in *black* and *red* the residues identified respectively as ML-DG and DG in panel (b). Calcium ions are shown as green spheres. Truncated curves in (**a**) and (**b**) represent peaks that were lost due to line broadening or overlap at higher temperatures.

Further analysis of structural changes was directed toward individual residues, using NMR. On the basis of {^1^H-^15^N} HSQC cross-peak intensities, we selected residues that could be tracked continuously over a suitable range of temperatures and classified each one according to whether the intensity diminished (decaying group, DG, *red*) or not (non-decaying group, NDG, *green*) (Fig. 5a and b). These changes in linewidth of NMR peaks are affected by protein tumbling, chemical exchange with the solvent and conformational exchange due to altered protein dynamics (see Discussion). Solely for the D145E variant, ten residues of the C-domain displayed a unique, biphasic behavior, with intensity increasing up to about 40 °C and then decreasing (L97, L98, D132, E134, D149, Y150, E152, E155, M157, K158). In accordance with nomenclature used for other proteins^31^, these were classified as mountain-like-DG (ML-DG, *black*) (Fig. 5b), and they represent residues that begin shifting to a different conformation as the temperature passes 30–40 °C. ML-DG residues of the C-domain are shown on the ribbon structure in Fig. 5c.

### Calcium binding to cTnC at different temperatures

**Figure 6:**
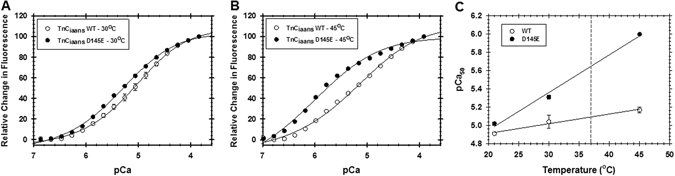
The increase in site II Ca^2+^ binding affinity with temperature is greater for D145E than WT. Proteins double-labeled at their native cysteines with the fluorescent probe IAANS were titrated with Ca^2+^ in the presence of Mg^2+^ at 30 °C (**a**) or 45 °C (**b**). Panel (**c**) summarises the changes in Ca^2+^ affinity (pCa_50_) from 21° to 45 °C, with a vertical dashed line to indicate pCa_50_ values extrapolated to 37 °C. Protein concentration was 1 µM. Excitation was at 330 nm and emission was monitored at 450 nm. Symbols show mean ± s.e.m. (n = 4).

The experiments described so far have shown that the ^1^H^N^ chemical shifts and other aspects of HcTnC D145E structure were markedly affected by temperature between 30° and 40 °C. Accordingly, we analysed the effect of temperature on the Ca^2+^ affinity of the mutant in solution. In isolated HcTnC labeled with the fluorescent probe 2-(4′-(iodoacetamido)anilino)naphthalene-6-sulfonic acid (IAANS), D145E had a higher affinity (by 0.11 log units) than WT for Ca^2+^ binding to the N-domain site at room temperature (21 °C)^11^. Here we show that this difference increased to 0.83 log units when the temperature was raised to 45 °C (Table 1). In other words, Ca^2+^ sensitivity at the N-domain increased 1.8 fold for the WT protein and 10 fold for the mutant, so that the difference in Ca^2+^ affinity between WT and mutant at the N-domain was much greater at 45 °C than at room temperature^11^. Figure 6a and b show the titrations for 30 and 45 °C, and Fig. 6c summarises the shift in Ca^2+^ affinity from 21° to 45 °C. By extrapolating between 30 and 45 °C, one can calculate that the Ca^2+^ affinity at 37 °C would be 3.8-fold higher for D145E than for WT.

**Table 1:** Ca^2+^ binding to HcTnC D145E and WT proteins labeled with IAANS increases with temperature, and the mutant is more susceptible.

HcTnC (°C)	pCa_50_	∆pCa_50_ (relative to 21 °C)	*n*_Hill_
WT (21°)	4.91 ± 0.01	—	0.90 ± 0.01
WT (30°)	5.04 ± 0.07	0.13	0.85 ± 0.01
WT (45°)	5.17 ± 0.03	0.26	0.67 ± 0.01
D145E (21°)	5.02 ± 0.02	—	0.88 ± 0.01
D145E (30°)	5.31 ± 0.02	0.29	0.81 ± 0.03
D145E (45°)	6.00 ± 0.01	0.98	0.71 ± 0.01

Proteins were labeled at Cys 35 and Cys 84 with IAANS, dialysed into fluorescence buffer containing EGTA, nitriloacetic acid, MOPS and KCl (*see Methods*) and adjusted to 1 µM before adding 1 mM MgCl_2_ and 1 mM DTT. After 10 min equilibration at the indicated temperature, proteins were titrated with CaCl_2_. pCa_50_ (−log of [Ca^2+^] for 50% of maximum fluorescence) and *n*_Hill_ are from Fig. 6A and B or (for 21 °C) from Pinto *et al*.^10^. Data reported at 21 °C were not tested against the others. Errors are ± s.e.m. (n = 4–5 at 21° and n = 4 at 30° and 45 °C).

pCa_50_ values at the same temperature (30°C or 45°C) and *n*_Hill_ values at 45°C are significantly different for the two proteins (p < 0.02).

pCa_50_ values for D145E are significantly different (p < 0.02) at 30° and 45°C.

n_Hill_ values for the same protein are significantly different (p < 0.02) at 30° and 45°C.

**Figure 7:**
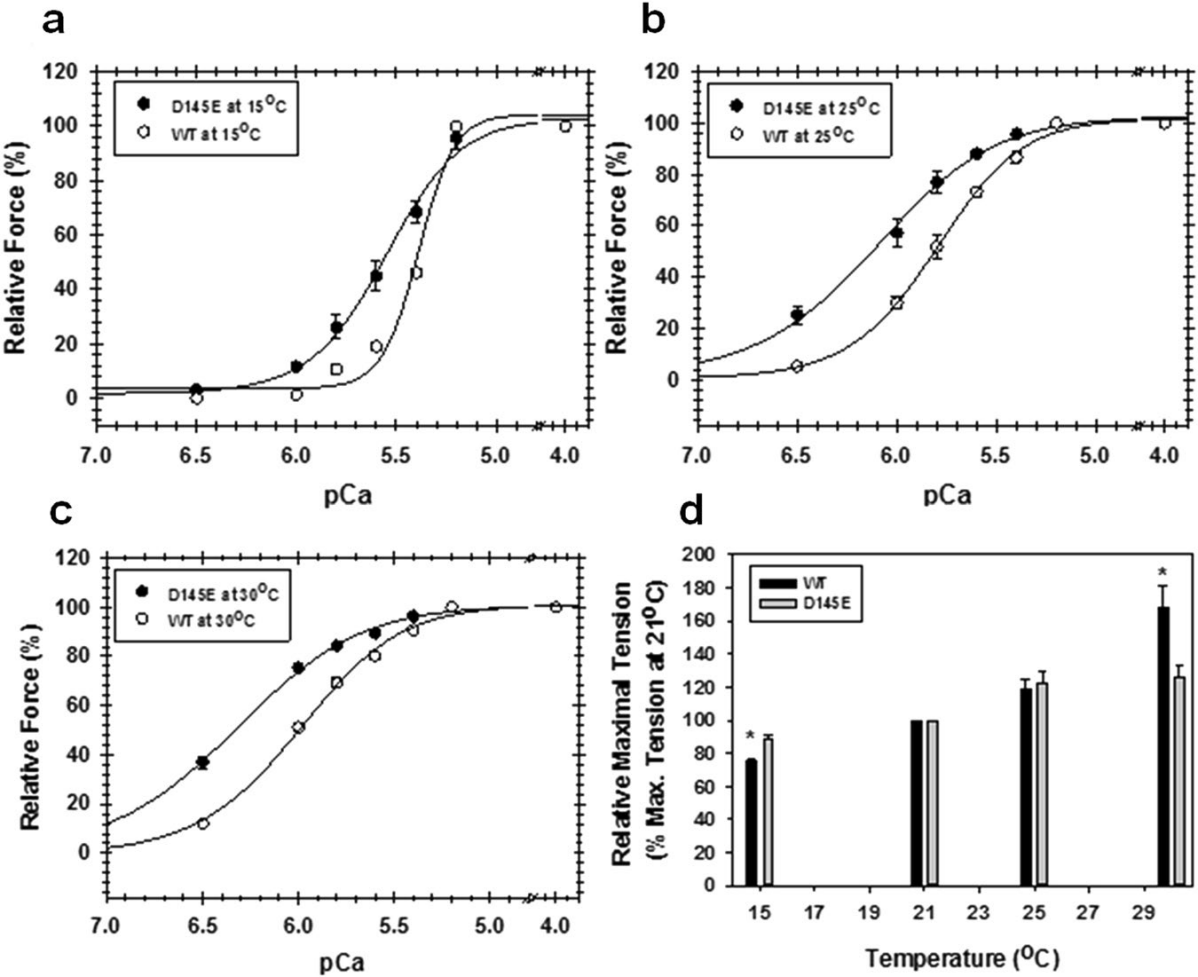
Temperature dependence for Ca^2+^ sensitivity and maximum force of cardiac skinned fibres is more pronounced following reconstitution with D145E cTnC. The Ca^2+^ sensitivity (pCa 8.0 to 4.0) of isometric force was measured at (**a**) 15° (**b**) 25° and (**c**) 30 °C in fibres depleted of native cTnC and reconstituted with HcTnC WT (○) or D145E (●). Ca^2+^ sensitivity was measured at all three temperatures on each fibre. Each curve is normalised to its own maximum (at pCa 4.0). (**d**) In a different set, each fibre was reconstituted with one isoform and maximal tension was measured at 21 °C and at one other temperature (*see Methods*). Data are normalised to the value at 21 °C. *p < 0.05 compared to WT at the same temperature. In (**a–d**), values shown are means ± s.e.m. (n = 4–5). In some cases error bars fall within the symbols.

The interaction of TnC with its partners in the Ca^2+^ regulatory system on the thin filament is an important determinant of Ca^2+^ affinity at the N-domain of cTnC^10, 11^. Thus we also tested the Ca^2+^ sensitivity at different temperatures in skinned fibres, where TnC operates in a complex environment that contains all the myofilament proteins. Fibres reconstituted with each isoform became more sensitive to Ca^2+^ with the elevation of temperature; once again, the effect with the mutant was more pronounced (Table 2 and Fig. 7). Because fibres are more fragile than isolated TnC^32^, in this case we could only cover a range of 15 °C, but the results were clear: from 15 to 30 °C the Ca^2+^ affinity of the fibres with mutant increased 8.1 fold, while the affinity for those with WT protein increased only 3.7 fold.

**Table 2:** Apparent affinity for Ca^2+^ increases more with temperature in cardiac skinned fibres reconstituted with HcTnC D145E.

	pCa_50_	∆pCa_50_ (relative to 20–21 °C)	*n*_Hill_
**WT**			
21 °C	5.66 ± 0.01	—	2.74 ± 0.19
15 °C	5.39 ± 0.01	−0.27	4.94 ± 0.36
25 °C	5.80 ± 0.03	0.14	2.07 ± 0.08
30°C	5.96 ± 0.04	0.30	1.65 ± 0.06
**D145E**			
21°C	5.90 ± 0.01	—	2.73 ± 0.17
15°C	5.51 ± 0.04	−0.39	2.44 ± 0.12
25°C	6.13 ± 0.06	0.23	1.56 ± 0.08
30°C	6.42 ± 0.02	0.52	1.32 ± 0.05

The Ca^2+^ sensitivity of isometric force was measured at 15°, 25° and 30° on each fibre, after extraction of endogenous cTnC and reconstitution with recombinant HcTnC, as described in Methods. pCa_50_ and n_Hill_ are from the experiments of Fig. 7a–c (n = 4–5) or (at 20–21 °C) from Landstrom *et al*.^8^ (n = 8). Data reported at 20–21 °C were not tested against the others. Errors are ± s.e.m.

For each protein, pCa_50_ values at 15 °C, 25 °C and 30 °C are significantly different from each other (p ≤ 0.022). Paired *t*-test was used.

pCa_50_ values at the same temperature (15 °C, 25 °C or 30 °C) are significantly different for the two proteins (p ≤ 0.01). Unpaired *t*-test was used.

In the last column, *n*_Hill_ values for D145E are significantly different from WT (p ≤ 0.005) at the same temperature (15 °C, 25 °C or 30 °C). Unpaired *t*-test was used.

In the last column, *n*_Hill_ values within each group (WT or D145E) are statistically different from each other (p ≤ 0.028). Paired *t*-test was used.

The slope (n_H_) of the HcTnC Ca^2+^-binding curves was also altered by temperature. The increase in temperature from 15 to 30 °C significantly reduced the cooperativity in both sets of fibres (p < 0.05). Fibres reconstituted with the mutant had less cooperativity than those with WT at each of these temperatures (Fig. 7 and Table 2). Experiments were not done above 30 °C because the fibres deteriorated too rapidly, as reported by others^20^. We note that Harrison and Bers found no further change in Ca^2+^ sensitivity of rabbit cardiac fibres when the temperature was raised from 29° to 36 °C^20^.

Since fibres exposed to 30 °C also suffer some deterioration^20^, we carried out control experiments for the effect of prolonged exposure to 30 °C. After 10 and 20 min, the maximal tension for fibres reconstituted with WT (Supplementary Fig. 1a) was more stable than in fibres reconstituted with the mutant (Supplementary Fig. 1b). Notably, the Ca^2+^-sensitivity values after prolonged exposure to 30 °C (Supplementary Fig. 1c and d) were not different statistically from the values extracted from Fig. 7. This result shows that deterioration of fibres exposed to a higher temperature (30 °C) did not affect the apparent Ca^2+^ sensitivity of the thin filament even though it decreased the maximal force. The same effect was previously shown in rabbit ventricular skinned fibres^20^.

Maximal force was also examined using only brief exposures to the higher temperatures in order to evaluate the short-term stability of the two isoforms on the thin filament (Fig. 7d). Raising the temperature potentiated the maximal force response in both cases, but at 30 °C, fibres with HcTnC WT were more robust, with maximal force reaching 168% of the original value (at 21 °C) while the fibres containing D145E developed only 126.5% of the original value.

## Discussion

In a previous report, Swindle and Tikunova analysed the C-domain changes wrought by three HCM mutations in isolated recombinant HcTnC^9^. The mutant D145E was particularly affected, with a greatly reduced Ca^2+^ affinity in the C-terminal domain. The authors concluded that this mutant could bind Mg^2+^ only at site III, failed to open upon binding Ca^2+^, and bound only weakly to the regulatory peptide (switch and inhibitory regions) of cTnI unless a great excess of Ca^2+^ (1 mM) was present. However, a comparison among progressively more complex model systems reconstituted with the D145E mutant has shown that the incorporation of other myofibrillar proteins (TnI, TnT, tropomyosin, actin and rigor cross-bridges) contributes significantly to its ability to regulate the thin filament at room temperature. As a result, in fact it exhibits only a mildly defective functional profile in reconstituted skinned fibres, where the response to Ca^2+^ is dominated by the N-domain^8, 11^. Thus, despite the remarkable array of dysfunctions in the C-domain, HcTnC D145E is capable of binding to myofibrils, reconstituting normal tensions and regulating full-fledged Ca^2+^ responses with only a slight departure from WT in Ca^2+^ off-rates from the N-domain site^8, 10, 11^. The question we have addressed is whether this is an accurate picture of its performance at higher temperatures, near the physiological range. A second question concerns interdomain communication: how does a defect in the C-domain produce a change in affinity for Ca^2+^ in the N-domain?

We have used two NMR tools related to structural transitions to focus our analysis of changes in the D145E isoform, measuring ^1^H^N^ temperature coefficients and ^1^H-^15^N cross-peak intensity changes over a range of temperatures. The first set of observations was analyzed in conjunction with the presentation of results from Figs 3 and 4. Here we discuss some of the factors that lead to changes in resonance intensities as a function of temperature (Fig. 5). The tumbling rate of the protein will always increase with temperature, making lines sharper and signals more intense. For amide hydrogens that are in conformational exchange (motions on a µs-to-ms timescale^33^) the lines can become broader (lower intensity) or sharper (higher intensity), depending on the relaxation parameter R_ex_, which is the exchange contribution to the observed R_2_ (R_2obs_ = R_2_ + R_ex_). The amplitude of R_ex_ depends on the exchange regime, the exchange rate (k_ex_) and the thermodynamic parameters of the conformational equilibrium. Larger values of R_ex_ lead to broader lines and a decrease in intensity.

In Fig. 5 we observed that the NDG residues (green lines) increased in intensity with the increase in temperature. They are responding to faster tumbling rates, or to smaller R_ex_. The DG residues (red lines), on the other hand, lost intensity with the increase in temperature. They are mostly responding to faster solvent exchange and are not involved in secondary structure. Some of them may also be increasing the exchange contribution (R_ex_).

More remarkable is the ML-DG group of residues, found only in the mutant. Between 15 and ~40 °C, intensities of these residues increased, followed by a decline at higher temperatures (~40 to 55 °C). This subgroup is certainly in conformational equilibrium between the native state and a higher-energy conformational state that was not present at the lower temperatures, and may be linked to an incipient unfolding event like that observed in the DG residues. Together with the CD data (Fig. 2), these observations on D145E structure reveal a more complex conformational equilibrium compared to the WT protein, and they also show that there are short-lived intermediates in the ML-DG population at 30–40 °C, involving ten residues in the C-domain.

In a related study of the WT and D145E proteins, experiments designed to measure Carr-Purcell-Melboom-Gill relaxation dispersion in the mutant identified a small, kinetically trapped excited state not found in the WT protein^18^. Flexibility of the D/E linker was demonstrably maintained.

Other studies of structural changes in cardiomyopathy-associated mutants of HcTnC have provided important information on flexibility, tertiary structure and interactions with troponin I, focusing primarily on the domain carrying the mutation^34, 35^. In terms of mechanism, the principal contribution of the present investigation has been to link the N-domain response to structural instabilities introduced by the C-domain mutation. As shown in Figs 2 and 3, the overall secondary structure of the WT and the mutant in the holo form changes very little, but the mutant melts more readily when heated. The melting curves represent the denaturation of full-length HcTnC, and it is not possible to say which domain has its stability more affected by the mutation with the CD assays. Based on melting curves for cardiac and skeletal TnC^4, 24, 36^, the C-domain dominates the signal and is less stable, but does not necessarily unfold first even in the WT proteins^24, 36^. However, since Ca^2+^ binding affinity for D145E C-domain is drastically reduced and only the curve for the Ca^2+^-bound mutant differs from WT, we infer that it is the C-domain that is most affected. The mass-spectrometry and bis-ANS experiments suggest that both C-domain sites bind Ca^2+^ poorly^18^, while site III can still bind Mg^2+^ ^9^. Based on CD data, the secondary structure of the Mg^2+^-loaded C-domain is similar for the WT and mutant proteins^11^. With regard to stability, the mutation renders the C-domain less stable than for WT, and this is reflected in the response of the N-domain when Ca^2+^ binds to site II. Thus temperatures near the physiological range (30° to 40 °C) significantly perturb the overall structure and function of D145E.

Alterations in the stability and function of the mutant could be related to lack of Ca^2+^ binding at site IV. Mutated residue 145 is located in site IV at the coordinating position z+. The exchange of aspartate for glutamate has been suggested as a destabilising element in the β-sheet structure that couples the two high-affinity sites but, as shown previously^11^, has only a small effect on the α-helical content overall. It is useful to compare the D145E mutation with D141A, analysed structurally and functionally by Putkey and co-workers^37, 38^. Like D145E, D141A abolished Ca^2+^ binding to site IV. Their data showed clearly that loss of site-IV Ca^2+^ binding had virtually no effect on Ca^2+^ sensitivity or maximum force in skinned fibres. In marked contrast to D145E, however, there were essentially no global effects of the D141A mutation on structure, as analysed in solution by NMR^37^. The β-strands between Ca^2+^-binding loops III and IV formed normally in the presence of Ca^2+^, and the signals from the aromatic residues of the hydrophobic core were virtually identical to those for the WT protein. These results are particularly interesting because the NMR experiments were carried out at 40 °C, very near the physiological range and close to where our experiments have shown the greatest effects with D145E. While the D141A mutation eliminated Ca^2+^ binding, it would likely also decrease electrostatic repulsion within a native-like site-IV conformation, whereas the D145E mutation would exacerbate electrostatic repulsion in the native-like conformation.

Multiple studies of different TnC species and domain fragments provide evidence for interdomain communication^4, 6, 15, 39^. How does the communication between N- and C-domains occur? The distance between the N- and C-domains of cTnC is about 48 Å^40^ and it has been suggested that the two domains may interact directly because of the bending of the flexible D/E linker in the troponin complex^17^. Conceivably, a mechanism based on flexibility of that link between N- and C-domains could be the main pathway for transmission of information from the C-terminus to the N-terminus. Nevertheless, even if the central D/E linker is in fact unfolded and mobile, the cTnI helix 1, which is embraced by cTnC C-domain, has several static segments. These might contribute to an additional pathway for signal transmission, one that passes along the core of the assembled thin filament^41^.

How do the results obtained with isolated proteins relate to those from skinned fibres? The incorporation of both recombinant HcTnC isoforms into skinned fibres increased the TnC response to both Ca^2+^ and temperature. The increase in Ca^2+^ affinity of cTnC upon incorporation into thin filaments is well documented, and can be attributed to interactions with other proteins of the regulatory complex as well as the presence of cross-bridges^10, 42^. An increase in Ca^2+^ sensitivity of force development with temperature is also expected^20, 32, 43^. Of particular interest in our experiments was the differential response of the mutant, where the increment in Ca^2+^ sensitivity was greater than for the WT protein at both 25 °C and 30 °C (Fig. 7b and c and Table 2). Since the only difference between the two sets of fibres was in cTnC and not in its partners on the thin filament, we can be confident that the differences in Ca^2+^ affinity seen with the mutant originate in the intrinsic structural problems of the isolated D145E protein.

It is noteworthy that the Ca^2+^ sensitivity of fibres containing the mutant at 30 °C is more than 3 times the value we observed earlier, at 20–21 °C ref. 8. This shift could have an important impact on relaxation kinetics. Little *et al*.^44^ have shown that cross-bridge detachment in cardiac myofibrils may limit the rate of relaxation up to about 20 °C, but not in a more physiological environment (35 °C and 100 µM ADP), where cross-bridges detach from actin nearly twice as fast as Ca^2+^ dissociates from cTnC. This means that near the physiological temperature, Ca^2+^ dissociation from WT cTnC will clearly be the rate-limiting step for cardiac muscle relaxation. Thus a 2.9 fold increase in Ca^2+^ affinity, as we have found for D145E at 30 °C, may retard relaxation and contribute significantly to diastolic dysfunction, a characteristic of the HCM phenotype^8^.

As noted above, it has already been demonstrated that the structural problems of the mutant affect its ability to interact with its partners^9^. In another assay, Marques *et al*. have now shown a tighter binding of the mutant to the thin filament^18^. Altered interactions may also account for the significant difference in cooperativity (n_H_ values) of the force *vs* pCa curves obtained in fibres with WT and mutant isoforms. In both cases the cooperativity decreased as the temperature was raised (Fig. 7 and Table 2), consistent with other reports for native cTnC in cardiac fibres from rabbit^20^ and rat^32^. In our experiments the cooperativity was generally lower (p < 0.05 at 15°, 25°, 30 °C) for fibres reconstituted with the mutant. D145E is located in the C-domain, which makes contact with TnT and thus indirectly with tropomyosin. Thus it is in a position to affect tropomyosin function, thereby altering the cooperativity within or between regulatory units.

The importance of events within functional regulatory units and the influence of strong cross-bridges can be appreciated in our data by comparing the low cooperativity of Ca^2+^ binding to the isolated cTnC subunits (n_H_ all < 1.0, Table 1) with the much steeper curves in fibres (n_H_ 1.3 to 5.0, Table 2). In fibres, the greater cooperativity at lower temperatures has been attributed to a reduced Ca^2+^ binding affinity for the N-domain sites caused by reduced force per cross-bridge together with a reduced number of cross-bridges^32^. This impact is transmitted along a chain of interactions between myosin binding sites on actin, Tm, TnT, TnI, and finally TnC – and would obviously be absent in the isolated protein. Evidently, any defects in a mutant TnC would add to the overall effect–specifically, in the case of D145E, reduced affinity of TnC N-domain for TnI^9^ and enhanced affinity for Ca^2+^ (Tables 1 and 2).

In conclusion, we report evidence of allosteric communication between the N- and C-domains, through changes in stability that involve backbone amide hydrogens and rather sharp conformational transitions between 30° and 40 °C. This concept may apply for other EF-hand proteins with two different classes of Ca^2+^ binding sites such as calmodulin. Instability distributed throughout the molecule is for the first time related to alterations in function, particularly Ca^2+^ sensitivity. An important next step will be to examine the mutant in the environment of a living cell^45^. Knowing the full range of effects caused by sarcomeric cardiomyopathic mutations will allow us to better understand the disease-associated phenotype and may help to design specific therapeutic interventions.

## Materials and Methods

### Protein expression and purification

The amino-acid substitution at residue 145 of human troponin C was inserted into HcTnC^8^. The expression and purification of HcTnC WT and D145E were carried out as described previously^8^. For use with skinned cardiac myofibrils, collected protein fractions were dialysed exhaustively against 5 mM NH_4_HCO_3_ and then lyophilised. Proteins were then resuspended and dialysed against pCa 8 solution (described below). For NMR, CD and fluorescence experiments, protein batches were maintained in solution at −80 °C, were not subjected to thawing and freezing cycles, and were not used after a month of storage. Aliquots of fresh purified samples were dialysed or diluted in the specific buffer and centrifuged at 10,000× *g*, 10 min, 4 °C; concentration was measured by the Lowry method and adjusted for each experiment.

### Resonance assignment

NMR data were acquired at 25 °C using a Varian Inova 600 MHz with a triple resonance cryogenic probe system at the Brazilian Biosciences National Laboratory (LNBio), Campinas, Brazil. Because significant changes were detected for the WT and D145E ^1^H-^15^N HSQC, we judged it necessary to obtain a set of three-dimensional spectra (HNCACB, CBCA(CO)NH, HN(CA)CO and HNCO) to complete the sequential HcTnC WT and D145E backbone assignment. The ^15^N,^13^C-labeled WT and D145E proteins were prepared as described previously^46^. Prior to NMR measurements, protein buffer was exchanged to 20 mM MOPS, 6 mM CaCl_2_, 1 mM MgCl_2_, 100 mM KCl, 10 mM DTT, pH 7.0 using a Vivaspin 20 10,000 MWCO (GE Healthcare Life Sciences) at 4 °C. Samples were prepared with a concentration range of 0.5–1 mM containing 10% D_2_O. The Computer Aided Resonance Assignment software (CARA 1.8.4, http://cara.nmr.ch/doku.php) was used as assignment platform. For thermal susceptibility experiments we used a Bruker Avance 500 MHz equipped with a probe heater at the National Center of Nuclear Magnetic Resonance Jiri Jonas (Rio de Janeiro, Brazil). ^1^H-^15^N HSQC experiments from WT and D145E were acquired at 15, 17.5, 20, 25, 30, 35, 40, 45, 50 and 55 °C and shift changes were monitored as a function of the temperature.

For chemical-shift perturbation (CSP) analysis, data processing was carried out using the NMRPipe software^47^. Data analysis was performed with CCPN (http://www.ccpn.ac.uk/) software using equation (1), and confirmed by careful analysis of the superposition spectra of WT and D145E.

1 $${CSP}={[{({\rm{\Delta }}{\delta }_{H})}^{2}+0.1{({\rm{\Delta }}{\delta }_{N})}^{2}]}^{1/2}$$

where Δδ_H_ and Δδ_N_ are the chemical shift variations between the WT and D145E of ^1^H and ^15^N, respectively.

^1^H chemical shifts as a function of temperature for each assigned residue were fitted using a linear regression, and the temperature coefficients were obtained from the slope, in ppb/°C. Nonlinear shifts were fitted using a second-order polynomial equation or two linear equations, as required. NMR assignment of {^1^H-^15^N}HSQC spectra at different temperatures was done by tracking the systematic shifts, using the assigned {^1^H-^15^N}HSQC at 25 °C as a reference. For CSP and thermal susceptibility experiments, only cross-peaks not involved in overlapped signals were included. After heating to 55 °C, thermal reversibility was checked by returning to 25 °C. WT and D145E spectra were totally reversible.

### CD spectroscopy

Far-UV CD spectra were collected in a Chirascan spectropolarimeter (Applied Photophysics) using a 1-mm-path quartz cell. Each protein was heated from 10 or 20 °C up to 90 °C at a rate of 1 °C/min, and the ellipticity was recorded at 222 nm every 1 °C. At 20° and 90 °C full spectra were recorded (200–260 nm, 50 nm/min) after temperature equilibration for 5 min. Three scans were averaged at each of these temperatures, and no numerical smoothing was applied. Immediately after reaching 90 °C, the temperature ramp was reversed in order to refold the protein. The optical activity of the buffer was subtracted from each protein spectrum. Molar ellipticities [*θ*] in degrees.cm^2^.dmol^−1^ were calculated using Equation (2)^48^:

2 $${[\theta ]}_{MRE}=\theta \frac{[0.1(\frac{MW}{n})]}{lc}$$

where *θ* is the measured ellipticity in millidegrees, *l* is the path length in cm and c the concentration in residue moles per litre. Protein concentration was 0.1 mg.mL^−1^. Protein concentrations were determined by the Lowry method using bovine serum albumin as a standard. The CD experiments were performed using buffers containing (in mM) 1 K_2_EGTA, 20 MOPS and 100 KCl at pH 7.0, plus for apo cTnC, no further addition; and for Ca^2+^/Mg^2+^-bound, 2.075 MgCl_2_ and 1.096 CaCl_2_ (yielding a free [Ca^2+^] of 10^−4^ M and free [Mg^2+^] of 2 mM). *T*_*m*_ values were obtained by taking the first derivative of the mean residue ellipticity (MRE)_θ=222_ *vs* temperature fitting. Data were fitted with a biphasic dose response function in OriginPro (Northampton, MA, USA):

3 $$y={A}_{1}+({A}_{2}-{A}_{1})[\frac{p}{1+{10}^{(\mathrm{log}x01-x)h1}}+\frac{1-p}{1+{10}^{(\mathrm{log}x02-x)h2}}]$$

where *A*_*1*_ and *A*_*2*_ are the bottom and top plateau values, logx01 and logx02 are the temperatures at the first and second midpoint transitions, *h1* and *h2* are Hill slopes and *p* a proportion factor.

### Ca^2+^ titration of isolated HcTnC

Both HcTnC isoforms were labeled with 2-(4′-(iodoacetamido)anilino)naphthalene-6-sulfonic acid (IAANS, Molecular Probes) at their native cysteines, Cys 35 and Cys 84; in this situation the signal from pCa 6.8 to 3.8 is a function of the N-domain sites^11^. Isolated IAANS-labeled HcTnCs were dialysed into fluorescence buffer containing 2 mM K_2_EGTA, 5 mM nitrilotriacetic acid, 120 mM MOPS, 90 mM KCl and pH 7.0. Before each titration, protein was adjusted to 1 µM, and 1.25 mM MgCl_2_ (0.45 mM free [Mg^2+^]) and 1 mM fresh DTT were added. Steady-state fluorescence measurements for Ca^2+^ binding at the N-domain of HcTnC_IAANS_ (WT or D145E) were performed in a four-cell, Peltier-controlled Jasco FP-8300 spectrofluorimeter where IAANS fluorescence was excited at 330 nm and emission was detected at 450 nm. The cuvette containing the protein was equilibrated for 10 min at the experimental temperature before starting the titration. Calcium titrations at 30 °C and 45 °C (pCa 7 to 3.8) were carried out as previously described for 21 °C ref. 11, but correcting the calculated pCa values for the higher temperatures using the program pCa Calculator^49^. The data obtained previously at 21 °C were imported for comparison. At 21 °C, free Mg^2+^ increased from 0.45 mM (at pCa 7) to 0.61 mM (at pCa 3.8). At 30° and 45 °C, the free Mg^2+^ was lower by 0.12 mM and 0.29 mM, on average, at each pCa value.

### Bis-ANS fluorescence

The bis-ANS fluorescence spectra were recorded at 20 °C on an ISSK2 spectrofluorometer (ISS, Inc.). Each protein was dialysed into fluorescence buffer containing 120 mM MOPS, 100 mM KCl and 2 mM K_2_EGTA (pH 7.0). Before each data set, 1 mM fresh DTT was added. For the holo state, Ca^2+^ and Mg^2+^ were added to obtain 0.1 mM free Ca^2+^ and 2 mM free Mg^2+^. Protein concentrations were 1 µM, mixed with buffer and 5 µM bis-ANS. Excitation was at 360 nm and emission at 400–600 nm.

### Skinned fibres

Cardiac fibre bundles were dissected from pig papillary muscle, skinned with Triton X-100 and prepared for recording as described^8^. Native cTnC was removed and replaced using a saturating concentration (109 µM) of HcTnC (WT or D145E) in relaxing solution. cTnC extraction and reconstitution were measured by recording residual tension at pCa 4.0, followed by pCa 8.0. The residual force, an index of remaining endogenous cTnC, was 16.9 ± 2.7% of P_0_ for fibres used in WT reconstitutions and 13.6 ± 1.2% of P_0_ for fibres with D145E (P > 0.05). Western blots have shown that 20% residual force at pCa 4.0 corresponds to an endogenous cTnC content of 14% and that ~70% recovery of P_0_ with HcTnC incorporation corresponds to a full complement of cTnC^50^.

The pCa 4.0 tensions recovered after reconstitution of TnC-depleted fibres were 69.9 ± 4.1% and 66.9 ± 2.0% of the original P_0_ at 21 °C (P > 0.05, N = 8) for WT and D145E, respectively. This corresponds to about 35 mN/mm^2^. Note that force recovery with the WT HcTnC reconstitution here was 10% higher than in Landstrom *et al*.^8^, most likely due to use of a ~4x higher concentration of cTnC to reconstitute the fibres. The reconstituted fibres were sequentially incubated with a range of [Ca^2+^] solutions containing 10^−8^ M to 10^−4^ M free [Ca^2+^], 1 mM free [Mg^2+^], 7 mM K_2_EGTA, 2.5 mM MgATP^2−^, 20 mM MOPS (pH 7.0), 20 mM creatine phosphate and 15 units/ml creatine phosphokinase; the ionic strength was adjusted to *I = *0.15 using K propionate. The pCa values were re-calculated for 15 °C, 25 °C and 30 °C using pCa Calculator^49^. The variations in free Mg^2+^, MgATP^2−^ and ionic strength were less than 10%. Data were analysed using equation (4):

4 $$ \% {\rm{Change}}\,{\rm{in}}\,{\rm{force}}=100\times {[{{\rm{Ca}}}^{2+}]}^{{\rm{nH}}}/({[{{\rm{Ca}}}^{2+}]}^{{\rm{nH}}}+{[{{{\rm{Ca}}}^{2+}}_{50}]}^{{\rm{nH}}})$$

where “[Ca^2+^_50_]” is the free [Ca^2+^] that produces 50% force and “nH” is the Hill coefficient. The initial force was measured at pCa 4.0 and 21 °C, then Ca^2+^ sensitivity of contraction and maximal force were recorded at 15° and 25 °C, and Ca^2+^ sensitivity was recorded at 30 °C. The data obtained previously at room temperature (20–21 °C) were imported for comparison. A separate set of fibres was used just for measurements of maximal force at 30 °C. In this case, a test for maximal force was performed at 21 °C, and then a Ca^2+^ curve ending at pCa 4.0 was carried out to provide a value for maximal force at 30 °C. This strategy was used because at 30 °C, considerable deterioration occurred in the maximal tension, different from the more stable tensions at 15 °C and 25 °C. The reconstituted P_o_ values at 15, 25 and 30 °C were normalised to the P_o_ recorded at 21 °C. In controls designed to evaluate fibre deterioration at 30 °C, the initial reconstituted P_o_ was recorded at 21 °C and then temperature was raised directly to 30 °C, where Ca^2+^ sensitivity was measured on the same fibre 0, 10 and 20 min after reaching 30 °C (Supplementary Fig. 1).

### Statistical analyses

All data were analysed for significance using Student’s *t-*test paired or unpaired at *p < *0.05 depending on the experimental design. The results are reported as means ± s.e.m.

## Electronic supplementary material

Supplementary Figure S1
