# Supplementary material for: Amide hydrogens reveal a temperature-dependent structural transition that enhances site-II Ca2+-binding affinity in a C-domain mutant of cardiac troponin C
Source: Sci Rep. 2017 Apr 6;7:691. doi: 10.1038/s41598-017-00777-6 (PMC5429600; doi:10.1038/s41598-017-00777-6)
Supplement: Supplementary file 1 — Supplementary Figure S1 [file 41598_2017_777_MOESM1_ESM.doc]

**SUPPLEMENTARY MATERIAL**

**Amide hydrogens reveal a temperature-dependent structural transition that enhances site-II Ca2+-binding affinity in a C-domain mutant of cardiac troponin C**

Tiago Veltri1,2,#, Guilherme A. P. de Oliveira3,#, Ewa A. Bienkiewicz1, Fernando L. Palhano2, Mayra de A. Marques3, Adolfo H. Moraes3, 4, Jerson L. Silva3, Martha M. Sorenson2, Jose R. Pinto1,*

1Department of Biomedical Sciences, Florida State University College of Medicine,1115 West Call Street, Tallahassee, FL 32306-4300, USA

2Instituto de Bioquímica Médica, Universidade Federal do Rio de Janeiro, Av. Carlos Chagas Fo 373, Cidade Universitária, Rio de Janeiro 21941-902, RJ, Brasil

3 Programa de Biologia Estrutural, Instituto de Bioquímica Médica, Instituto Nacional de Biologia Estrutural e Bioimagem, Centro Nacional de Ressonância Magnética Nuclear Jiri Jonas, Universidade Federal do Rio de Janeiro, Rio de Janeiro, Brasil.

4Departamento de Química, Instituto de Ciências Exatas, Universidade Federal de Minas Gerais, Brasil.

#Co-first authors


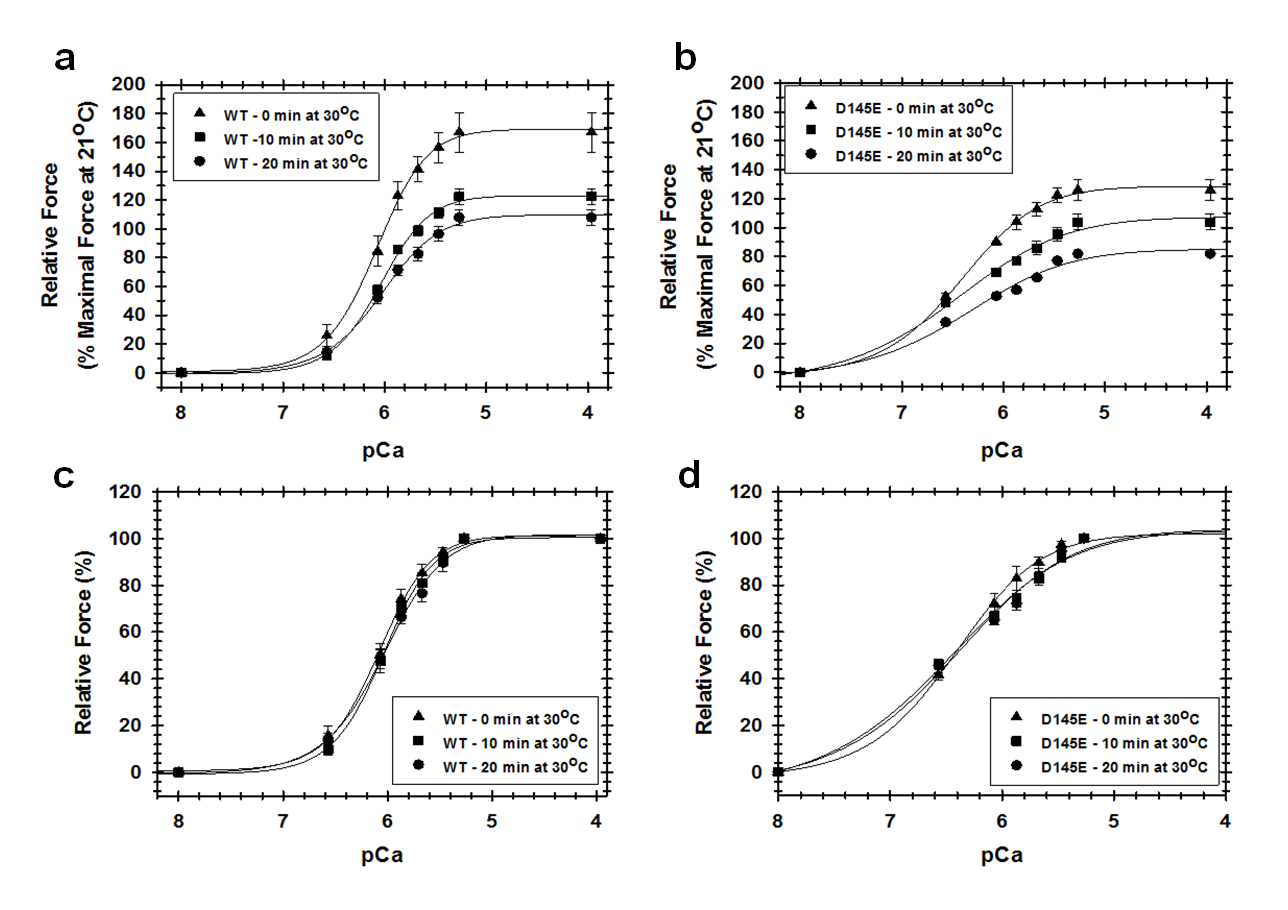
**Supplementary Figure S1.** **Prolonged exposure to 30°C causes a decrease in force but no loss of Ca2+ sensitivity in cardiac fibres.** Skinned fibres were reconstituted with HcTnC WT **(a)** or D145E **(b)**, maximally activated at 21°C (pCa 4.0), and returned to relaxing solution; the temperature was then raised to 30°C and maintained for pCa-force curves after 0 (▲), 10 ( ■ ) and 20 ( ● ) min at 30°C. Each curve is normalised to the original maximum value at 21°C. Figures **(c)** and **(d)** show the curves of (a) and (b) normalised to their own maxima. Values are averages ± s.e.m. (n = 3-4), and the pCa_50_ values were not significantly different (p > 0.05) at 0, 10 and 20 min.
